# Don't be afraid of black holes: Vacuum sponge and vacuum stent treatment of leaks in the upper GI tract—a case series and mini-review

**DOI:** 10.3389/fsurg.2023.1168541

**Published:** 2023-05-03

**Authors:** Christian Schäfer

**Affiliations:** Department of Gastroenterology, Robert-Bosch-Hospital, Stuttgart, Germany

**Keywords:** endoscopic vacuum therapy (EVT), cSEMS, esophageal leak, anastomotic leak (AL), OTSC (over-the-scope clip), esophageal stricture

## Abstract

The treatment of leaks in the upper gastrointestinal tract has been revolutionized by the advent of covered self-expanding metal stents (cSEMS), endoscopic vacuum therapy (EVT), and recently, vacuum stent therapy (VST). In this retrospective study, we report the experiences with EVT and VST at our institution.

**Patients and methods:**

Twenty-two patients (15 male, 7 female) with leaks in the esophagus, at the esophago-gastric junction or anastomotic leaks underwent EVT by placing a sponge connected to a negative pressure pump into or near the leak. VST was applied in three patients.

**Results:**

EVT led to closure of the leak in 18 of 22 Patients (82%). In 9 patients (41%), EVT was followed by application of a cSEMS. One patient (5%) died during the hospital stay due to an aorto-esophageal fistula near the leak, four others (18%) due to underlying disease. The stricture rate was 3/22 (14%). All three patients in whom VST was applied had closure of the leak and recovered. Reviewing the literature, we identified sixteen retrospective series of ten or more patients (*n* = 610) with an overall closure rate for EVT of 84%. In eight additional retrospective observations, a comparison between the efficacy of EVT and cSEMS therapy was performed that revealed a success rate of 89% and 69%, respectively (difference not significant, chi-square test). For VST, two small series show that closure is possible in the majority of patients.

**Conclusion:**

EVT and VST are valuable options in the treatment of leaks in the upper gastrointestinal tract.

## Introduction

Prior to the introduction of vacuum sponge or stent therapy, perforations or leaks of the upper GI tract were associated with a high risk of mortality or morbidity and often required divertive surgery (1–3).

The majority of leaks occur after oncological resection of the esophago-gastric junction or esophagus. Less common are iatrogenic perforations (e.g., after forced insertion of gastric tubes, endoscopes or transesophageal echocardiography probes into a narrowed esophagus or esophageal diverticulum) or spontaneous tears, such as Boerhaave ruptures.

Small perforations may be handled by application of a row of several through-the-scope (TTS) clips. Over-the-scope clips (OTSC) might be effective for perforations ≤1 cm provided that the clip grasps not only the mucosa, but also the muscularis propria of two opposite edges of the leak.

Larger leaks in the tubular esophagus may be temporarily “sealed” by covered self-expanding metal stents (cSEMS) applied *via* upper GI endoscopy thus preventing bacteria from ingested food and saliva from migrating into the mediastinum. This method, which allows regular oral food intake, is optimal for perforations of the tubular part of a non-dilated, non-operated esophagus, whereas coverage of anastomotic leaks, e.g., after esophagectomy, may be insufficient due to the wider lumen of the adapted part of the stomach so that the cSEMS might lose contact to the wall.

Endoscopic vacuum sponge therapy (EVT) is another approach to treat leaks. Previously applied to rectal leaks, it has been adapted to the upper GI tract (4–6). A sponge positioned in the leakage cavity and connected to a negative-pressure pump allows continuous debridement and accelerates the granulation and closure of the wound. For smaller defects, it might be sufficient to place the sponge into the lumen next to the leak. The sponge needs to be exchanged 2–3 times per week. During vacuum sponge therapy, enteral feeding *via* a nasoduodenal tube is possible, whereas oral food intake is not.

A more recent approach is the insertion of a covered stent surrounded by a small layer of sponge material connected to a transnasal suction tube (vacuum stent therapy, VST) (7, 8). With this combination of cSEMS and EVT technology, patients may be allowed to eat.

Whereas a cSEMS is easy to place, the vacuum-based technologies, EVT and VST, require advanced endoscopic skills and are only successful in a setting of good interdisciplinary cooperation with surgical and ICU partners. In addition, nutritional and anti-infective therapy should be optimized, and last but not least, the patient should be willing to undergo serial endoscopies and to accept continuous transnasal suction, which may occasionally last for several weeks.

The following case series illustrates the efficacy and the restraints of vacuum sponge and vacuum stent therapy.

## Methods

From our endoscopic records, we identified patients with leaks in the upper GI tract that were treated either with EVT or VST at the Robert-Bosch-Krankenhaus, Stuttgart, Germany, between 2010 and 2022.

### Endoscopic vacuum therapy (EVT)

From 2010 to 2014, we used a self-made sponge device derived from the Endosponge® rectal vacuum therapy set (B. Braun, Melsungen, Germany). The cylindrical sponge that had been trimmed to the size of the leakage cavity was removed from the original suction tube, because the latter was too short for intraesophageal placement. The tip of a nasogastric tube that had been inserted transnasally and led out through the mouth was sutured into the sponge. The surgical thread at the distal end of the sponge was knotted several times and grasped outside the patient using a gastroscope armed with a forceps. Under endoscopic guidance the sponge was placed into the leakage cavity of the upper GI tract. After initiating suction *via* the tube [minus 125 mmHg, using an electric vacuum pump (4)], the gastroscope could be removed. The sponge was exchanged three times per week.

In recent years, a simplified system with a smaller sponge attached to an extra-long plastic tube (Esosponge®, B. Braun, Melsungen, Germany) was used that could be placed *via* an overtube. The gastroscope armed with the overtube was advanced into the leakage cavity. After withdrawal of the scope, the overtube was left with the tip in the leakage cavity. Using a pusher, the sponge was advanced to its final position. Once the overtube and the pusher had been removed, the suction tube was deviated transnasally and connected to the vacuum pump. Continuous suction was applied (minus 125 mmHg) and the position of the sponge was controlled by endoscopy. The sponge was renewed three times per week (i.e., maximum intervals of three days), since longer intervals led to increased clogging and ineffectivity of the vacuum sponge.

### Vacuum stent therapy (VST)

The Vac-Stent® (Microtech, Düsseldorf, Germany) is a covered self-expanding stent (interior lumen diameter 12 mm) and surrounded by a 5 cm long vacuum sponge layer attached to a suction tube (7). The device can be introduced transorally like a standard metal stent. Subsequently, the suction tube needs to be redirected through the nose. Initial suction was applied at −125 mmHg, and then lowered to −75 mmHg. After 7 days, the stent was removed. If there was still a visible leak, the therapy was repeated.

### Additional measures for patients undergoing EVT or VST

In most patients, pleural effusions were drained by thoracic tubes, since anastomotic or other leaks usually communicate with the thoracic cavities. If more than one thoracic tube was required, each tube was connected to an independent suction device to avoid inadequate drainage. If there were signs of infection, antibiotic therapy was initiated. Adequate nutrition was provided by enteral feeding *via* a nasoduodenal or nasogastric tube that was placed prior to EVT. Sometimes parenteral nutrition was applied. In VST peroral feeding was allowed.

Optimization of vacuum therapy: In standard EVT, excess luminal fluid resulting from saliva or regurgitation of gastric fluid may impede the efficacy of suction. In some patients with excess saliva production, a transdermal scopolamine patch was applied. If regurgitation of excessive gastric fluid was thought to impede the vacuum effect of the sponge, an additional Esosponge® that was connected to a separate vacuum pump was positioned in the stomach. In one case with additional sponge, the patient received a feeding jejunostomy to enable enteral feeding.

### Statistics

For the evaluation of the retrospective studies comparing EVT vs. cSEMS, chi-square test was used.

## Results

### Endoscopic vacuum therapy

In the past 13 years, 22 patients, 15 male and 7 female, with a mean age of 67.8 years were treated with EVT. Four of these patients had been referred from other hospitals to our institution.

Most leaks were postsurgical (*n* = 14), occurring after resection of cancer of the tubular esophagus, the esophagogastric junction or the stomach, other patients (*n* = 4) developed leaks after endoscopic procedures, e.g., dilations, or forced placement of a nasogastric tube after hiatal hernia repair, or had a spontaneous rupture of the esophagus due to increased pressure (Boerhaave rupture) or inflammation (*n* = 4). In each individual patient, the decision to apply EVT was made jointly by the endoscopist (gastroenterologist) and the abdominal (or thoracic) surgeon.

In some patients, other leakage therapies had been tried without success, like the application of a large over-the-scope clip (OTSC, *n* = 3), placement of a covered self-expanding metal stent (cSEMS, *n* = 2) or surgical redo (*n* = 2). EVT usually started out with intracavitary sponge placement. Only two patients received intraluminal sponge therapy from the beginning because of a very small opening of the leak. The duration of EVT is reflected by the number of procedures ranging from 3 to 23 with a median number of 6 procedures. The majority of the patients (*n* = 18) received unilateral or bilateral thoracic drains to reduce pleural effusions.

Nine of 22 (41%) patients received a cSEMS as sequential therapy, for various reasons: In three patients, EVT failed, in one patient a stricture was opened by stent insertion, one patient had a bleeding aortoesophageal fistula. In the other four patients, cSEMS was used to cover minor residual blindly ending fistulae after successful EVT in order to reinstitute oral intake. In two patients (9%) additional surgery was necessary (surgical removal of solid food from the thoracic cavity).

Successful leakage closure—with or without sequential cSEMS—was achieved in 18 of 22 patients (82%).

In four patients (18%) the leakage could not be closed, i.e., we observed either no reduction in cavity size or fistula complications. One of these patients (aged 76 years, but without any additional risk factors) with a continuing anastomotic leak after 18 sponge placements received a cSEMS that allowed peroral nutrition and discharge from the hospital in a stable condition. The other three patients died within a short period (<2 months) after unsuccessful leakage treatment: One patient with an anastomotic leak developed fatal bleeding due to an aorto-esophageal fistula after five sponge placements that could not be stopped by cSEMS treatment. Two patients with persistent leaks had significant comorbidity that led to ventilatory and finally multiorgan failure (one patient with an anastomotic leak who had severe COPD and one patient with a spontaneous leak due to pleural empyema who had morbid obesity with a BMI > 40).

In addition to these three fatalities, two other patients died despite successful EVT due to septic port infection and progressive duodenal cancer, respectively.

Three patients (14%) developed a stenotic stricture that was successfully treated either with balloon dilation or, as mentioned above, placement of a removable cSEMS.

### Vacuum stent therapy

VST was applied in three patients, one with a perforation due to transesophageal echocardiography, and two with postoperative anastomotic leaks. In two patients, EVT and/or cSEMS had been tried unsuccessfully beforehand. In these two patients, the duration of VST had to be extended to 2 weeks requiring an exchange of the vacuum stent after the first week. Closure was achieved in all three patients.

## Discussion and review of the literature

The series presented in this paper confirms previous observations that EVT may substantially contribute to the healing of life-threatening leaks of the esophagus and the esophagogastric junction. Our findings are comparable to retrospective data from other groups.

### EVT case series

Table 1 summarizes several publications of case series of 10 or more patients, in whom EVT was applied. As in our study, most of these patients were male and suffered from postoperative anastomotic leaks. In our and other series, closure rates around 80% were achieved. Not only by us, but also by half of the other groups, EVT was combined with sequential cSEMS placement.

**Table 1:** EVT in patients with leaks in the upper gastrointestinal tract. Case series (≥ 10 patients).

Reference	Patients (*n*)	Male:Female	Etiology: anastomotic/iatrogenic/spontanous	Closure rate	Sequential cSEMS therapy	Surgical revision	Stricture	Short term mortality*
(9)	35	23:12	21/7/7	32/35	0	15	1	2
(10)	10	5:5	5/4/1	7/10	0	1	1	3
(11)	21	15:6	11/8/2	19/21	0	9	1	1
(12)	52	37:15	39/9/4	49/52	1	0	4	5
(13)	77	51:26	59/12/6	60/77	21	5	nd	12
(14)	12	12:0	12/0/0	8/12	0	1	1	1
(15)	13	6:7	2/8/3	5/13	0	3	0	1
(16)	20	20:0	20/0/0	19/20	0	0	7	0
(17)	22	17:5	22/0/0	19/22	0	1	3	0
(18)	30	20:10	23/7/0	25/30	2	3	4	2
(19)	23	20:3	23/0/0	19/23	3	0	0	3
(20)	20	19:1	20/0/0	19/20	4	0	0	1
(21)	92	59:33	92/0/0	80/92	4	24	12	6
(22)	102	72:30	69/9/24	88/102	9	26	10	7
(23)	26	20:6	26/0/0	13/26	11	4	3	0
(24)	55	46:9	55/0/0	49/55	0	5	0	4
**Total**	**610**	**442:168**	**499/64/47**	**511** **/** **610**	**55**	**97**	**47**	**48**
**Percent**	** **	**72%M**	**82%/10%/8%**	**84%**	**9%**	**16%**	**8%**	**8%**
Own series	22	15:7	14/4/4	18/22	9	2	3	1/22^#^ (4/22)°
Percent		68%:32%	64%/18%/18%	82%	41%	9%	14%	5%^#^ (18%)^°^

* Short term mortality: varying definitions, mostly in-house mortality.

^#^ Mortality in the context with anastomotic leakage.

° Mortality due to underlying disease.

Serious adverse events due to EVT were rarely reported by others. In our series, one patient developed an aorto-esophageal fistula with fatal bleeding that could not be stopped by cSEMS. In this case, non-adequate debridement or EVT itself might have contributed to the erosion of the aortic wall. Otherwise, the mortality rate in our series reflects demographic factors and severe comorbidities (old age, COPD, extreme obesity, progressive cancer).

A frequent chronic complication is the development of stenotic strictures. In our group, 3 out of 23 patients had to be treated by repeated balloon dilation or cSEMS insertion. This is comparable to the average stricture rate reported by others. Other complications, such as airway leaks, were not observed.

Early diagnosis of leaks and immediate action after the diagnosis may significantly improve the outcome of EVT and reduce in-hospital mortality. This is suggested by a retrospective study in 156 patients (not included in Table 1) with leaks of various etiologies comparing early experience with EVT with an optimized concept in recent years showing a significant reduction of the 30 day in-hospital mortality and improvement of leakage resolution (25). In the latter group the time gap between diagnosis of leakage and start of EVT had been significantly shortened compared to the early period (0.3 vs. 7.2 days).

### Retrospective comparison EVT vs. cSEMS

Is EVT better than or equal to cSEMS therapy in patients with esophageal and esophagogastric leaks? So far, only retrospective comparisons are available, as listed in Table 2. Most of these evaluations dealt with patients who developed anastomotic leaks after resective surgery for malignant tumors of the esophago-gastric junction or the esophagus.

**Table 2:** Retrospective comparisons of cSEMS vs. EVT.

Reference	Group	Patients (*n*)	Male:Female	Etiology: anastomotic/iatrogenic/spontanous	Closure rate	Treatment Switch (EVT ≥ cSEMS or cSEMS ≥ EVT)	Surgical revision	Stricture	Mortality
(26)	EVT	32	28:4	30/1/1	27/32	3	1	3	5
	cSEMS	39	30:9	31/6/2	21/39	0	3	11	11
(27)	EVT	17	nd	17/0/0	nd	nd	0	nd	2
	cSEMS	12	nd	12/0/0	nd	nd	2	nd	5
(28)	EVT	15	14:1	15/0/0	14/15	0	0	nd	1
	cSEMS	30	21:9	30/0/0	19/30	7	4	nd	8
(29)	EVT	7	5:2	7/0/0	7/7	0	0	nd	—
	cSEMS	11	9:2	11/0/0	7/11	1	3	nd	—
(30)	EVT	34	29:5	35/0/0	30/35^#^	6	0	1	3
	cSEMS	76	63:14	76/0/0	55/76^#^	7	0	5	11
(31)*	EVT	30	27:3	30/0/0	28/30	nd	nd	1	5
	cSEMS	14	11:3	14/0/0	13/14	nd	nd	5	1
(32)	EVT	13	12:1	13/0/0	12/13	nd	5	nd	5
	cSEMS	7	5:2	7/0/0	6/7	nd	1	nd	1
(33)	EVT	9	5:4	9/0/0	8/9	1	8	nd	0
	cSEMS	5	4:1	5/0/0	5/5	0	5	nd	0
**Total**	**EVT**	**157**	**120:20 (86%M)**	**155/1/1**	**126/141 (89%)**	10	14	**5/97 (5%)**	**21/150 (14%)**
	**cSEMS**	**194**	**143:40 (78%M)**	**186/6/2**	**126/182 (69%)**	15	18	**21/129 (16%)**	**37/183 (20%)**
Chi- square			n.s.		n.s.			*p* = 0.02	n.s.

nd, not explicitly detailed.

*Includes additional data from (34).

^#^ Closure rate calculated according to final therapy for patients who had been switched to the other group.

Despite the fact that each retrospective study used different criteria and definitions, it is obvious that EVT is at least non-inferior to cSEMS therapy, the reported numbers suggest even a slightly higher closure rate and a lower mortality rate, although this difference is not significant. The only significant difference was found for the stricture rate that was higher in the cSEMS group. A caveat for these retrospective studies is that patients of different eras may have been compared. For the future, sufficiently powered prospective randomized studies are needed that also allow subgroup analyses for different etiologies and locations of the leaks.

### Experiences with VST

A recent development is the vacuum stent therapy (VST) combining cSEMS and vacuum sponge technology. In our hospital, the experience is limited, but all three patients who have been treated with this method had closure of their leak and recovered. Although the effective length of the sponge material around the shaft of the vacuum stent is relatively short (5 cm), lesions that were not too large could be effectively sealed.

In the pilot study reported by Lange et al. (7), three patients with different etiologies (anastomotic leak, Boerhaave tear, one patient with a migrated LINX anti-reflux device which had been removed surgically and left a postoperative transmural opening) showed that VST induced granulation and closure of the gap.

Another retrospective report on a series of 10 patients published by Chon et al. confirmed the effectiveness of VST (8). Out of ten patients (five with anastomotic insufficiencies, five of other origin incl. Boerhaave syndrome) seven were treated successfully by VST. In the remaining three patients with VST failure, two were switched to EVT and one had to undergo esophagectomy.

These two reports, along with our experience, show that VST is technically feasible and leads to closure of tears or leaks in the esophagus or at the esophageal junction in most, but not all patients. Possibly, technical improvements, e.g., longer stents, may be more effective in covering larger leaks.

In summary, the therapeutic strategies developed for leaks of the esophagus and the esophagogastric junction have dramatically improved the clinical outcome. However, the experience is based on retrospective non-randomized observations. Larger prospective randomized comparative trials are needed to determine the optimal strategy for leaks of different etiologies and locations.
